# Programmable and Flexible Fluorochromic Polymer Microarrays for Information Storage

**DOI:** 10.1021/acsami.2c02242

**Published:** 2022-05-31

**Authors:** Hongyan Xia, Yuguo Ding, Jingjing Gong, Annamaria Lilienkampf, Kang Xie, Mark Bradley

**Affiliations:** † State Key Laboratory of Precision Electronic Manufacturing Technology and Equipment, School of Electromechanical Engineering, Guangdong University of Technology, Guangzhou, Guangdong 510006, China; ‡ EaStCHEM School of Chemistry, University of Edinburgh, Edinburgh EH9 3FJ, United Kingdom

**Keywords:** photoresponsive, fluorochromic, polymer microarray, Förster resonance energy transfer, information storage

## Abstract

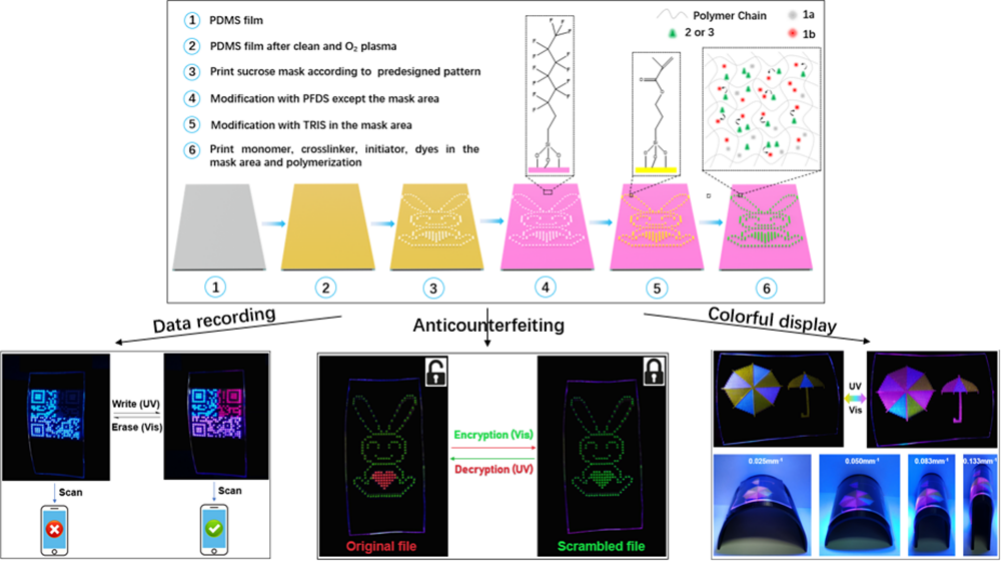

Photoresponsive fluorochromic materials are regarded as an effective means for information storage. Their reversible changes of color and fluorescence facilitate the storage process and increase the possible storage capacity. Here, we propose an optically reconfigurable Förster resonance energy transfer (FRET) process to realize tunable emissions based on photochromic spiropyrans and common fluorophores. The kinetics of the photoisomerization of the spiropyran and the FRET process of the composite were systematically investigated. Through tuning the ratios of the acceptor spiropyran and donor fluorophore and external light stimuli, a programmable FRET process was developed to obtain tunable outputs. More importantly, flexible microarrays were fabricated from such fluorochromic mixtures by inkjet printing (230 ppi) and the dynamic FRET process could also be applied to generate tunable fluorescence in ready-made microstructures. The flexible patterns created using the microarrays could be used as novel optically readable media for information storage by altering the composition and optical performance of every feature within the microarray. A key aspect of information storage such is anti-counterfeiting, and these colorful displays can be fabricated and integrated in a simple and straightforward system. The reliable fabrication and programmable optical performances of these large-scale flexible polymer microarrays represent a substantial step toward high-density and high-security information storage platforms.

## 1 Introduction

Society is entering an era of information and data, but how to store the vast amount of information efficiently has already become a major challenge.^1^ Compared with magnetic and electronic information storage technologies based on semiconductor materials, optical information storage exhibits the merits of low cost, high storage density, fast speed, easy portability, and low power consumption.^2,3^ Photoresponsive fluorochromic materials that can switch and modulate their fluorescence and color reversibly upon external light stimuli are very promising in optical information storage.^4−6^ Different from previous optical information storage such as digital versatile discs (DVDs), compact discs (CDs), and Blu-ray discs that are already replaced by solid-state memory, photoresponsive materials can well realize information recording and readout because they can undergo a series of reversible changes in certain physical and chemical properties in response to light stimulus.^7^ Information (re)writing and erasing,^8^ encryption and decryption,^9^ anti-counterfeiting,^10^ and displays^11^ based on photoresponsive fluorochromic materials can be ″read″ directly by their change of color and fluorescence, thus offering great convenience for optical information storage.^12,13^ Light makes photoresponsive fluorochromic materials easier to realize and put into practice than many other approaches; indeed, the rapid, accurate, and remote spatiotemporal resolution enabled by photomodulation will ensure a smooth realization for information storage.^14^ In addition, light can modulate the color and emission of photoresponsive materials precisely, preventing optical information recording and readout from being stolen or counterfeited. Also, organic photoresponsive fluorochromic materials exhibit unique advantages due to their high flexibility, light weight, broad spectral coverage, and compatibility with large-area solution processing techniques such as inkjet printing in comparison with their inorganic counterparts.^15^ One of the most commonly used strategies to obtain photoresponsive fluorochromic materials is through Förster resonance energy transfer (FRET) between functional photochromic units (energy acceptors) and fluorophores (energy donors).^16^ The reversible isomerization of photochromic units leads to light-based and dynamically tunable concentrations of the isomers, which can be used to modulate the stoichiometric ratio of the donor and acceptor, resulting in tunable FRET and output emissions of the composite system.^17−19^ Such a system offers the potential for high-level security and broader information processing and storage applications through the manipulation of the FRET process. Most of the FRET processes in previous works were carried out in solutions, even if some were in nanoparticles, these particles were still dispersed or dissolved in solutions, which were used as inks and written or printed on papers when applied for information storage. The capacity and modulation accuracy of the information storage are limited. Thus, the development of modulation for the FRET process in solid microscale, capable of rapidly switching emissions of each microunit in microstructures, is of great significance in the increasement of the modulation accuracy and density of information storage.

Limited and random outputs and distributions of emissions cannot meet the development of miniaturization and integration of optoelectronic components and devices, and well-organized and precisely controlled integration and assembly are urgently needed.^20^ Optical microarrays, which can integrate multifunctional materials together optically and regularly, will enhance the photonic properties and satisfy future optical integrated applications, such as information storage.^21,22^ Also, the ordered optical microarray provides an effective approach to boost writing/reading throughputs as it can increase information storage capacity and modulation accuracy. As such, it has been reported that the optical microarray has a potential to change the current magnetization-based approach for big data storage to the use of optical discs, with each microunit in the microarray having the ability to record and read information to maximize throughput.^23^ Inkjet printing is a mask-free patterning technique that can be used for the precise fabrication of optical microarrays in a controlled manner, with the advantages of rapid noncontact processing at low cost and inherent scalability.^24−26^ This method can utilize a variety of ″ink solutions″ with many nozzle formats, allowing direct deposition onto substrates according to predesigned patterns accurately.^27^ In addition, the morphology, size, and spacing of the printed features on the microarrays can be readily adjusted with precise alignment and position over a very large area.^28^ Inkjet printing thus provides a facile technology to deposit a wide variety of materials on various substrates in well-defined patterns.^29^

Spiropyrans, the typical photochromic compounds, have been extensively introduced into various functional materials due to their reversible photochemical interconversion between two isomers with distinct properties upon ultraviolet light (UV) and visible light (vis) irradiation.^30−32^ Herein, spiropyran **1** and two fluorophores (5(6)-carboxyfluorescein (**2**) and disodium 2,2′-[biphenyl-4,4′-diyldiethene-2,1-diyl]dibenzenesulfonate (**3**)) were integrated together to construct the photoresponsive fluorochromic materials through dynamic and tunable FRET process. Under 365 and 470 nm light irradiation, spiropyran **1** isomerizes between the ring-opened form (merocyanine, **1b**) that is fluorescent and the ring-closed form (spiro, **1a**) that is nonfluorescent. When mixed with a fluorophore, FRET will only occur between the fluorophore and the spiropyran isomer **1b** (acceptor). This FRET efficiency can be modulated by adjusting the ratio of the two isomers of spiropyran through different levels of light irradiation, resulting in different colors and fluorescence of the mixtures. What’s more, flexible and large-scale microarrays of the above photoresponsive fluorochromic materials with a controllable arrangement and specific functionalities on polydimethylsiloxane (PDMS) substrates by inkjet printing were obtained, with a resolution of 230 ppi. FRET was carried out in the solid, well-designed, microstructures, and the emissions of each microunit in microstructures can be regulated. Information writing and erasing, encryption and decryption, and display can be realized using the dynamic and colorful flexible microarrays. Owing to the versatility, compatibility, and convenient processing capability of the programmable fluorochromic polymer microarray, they could be applied in almost all aspects of information storage. This system is easy to fabricate without complex and troublesome synthesis. Every feature within the microarrays can be used as a pixel, while every individual pixel can be designed and modulated. Such multiple dynamic emission states can potentially carry high-levels of information. This precise adjustment at the microscale will greatly improve the modulation accuracy and information storage capacity in ultracompact photonic components.

## 2 Results and Discussion

### 2.1 Dynamic FRET Processes Based on the Photochromic Moiety

**Figure 1:**
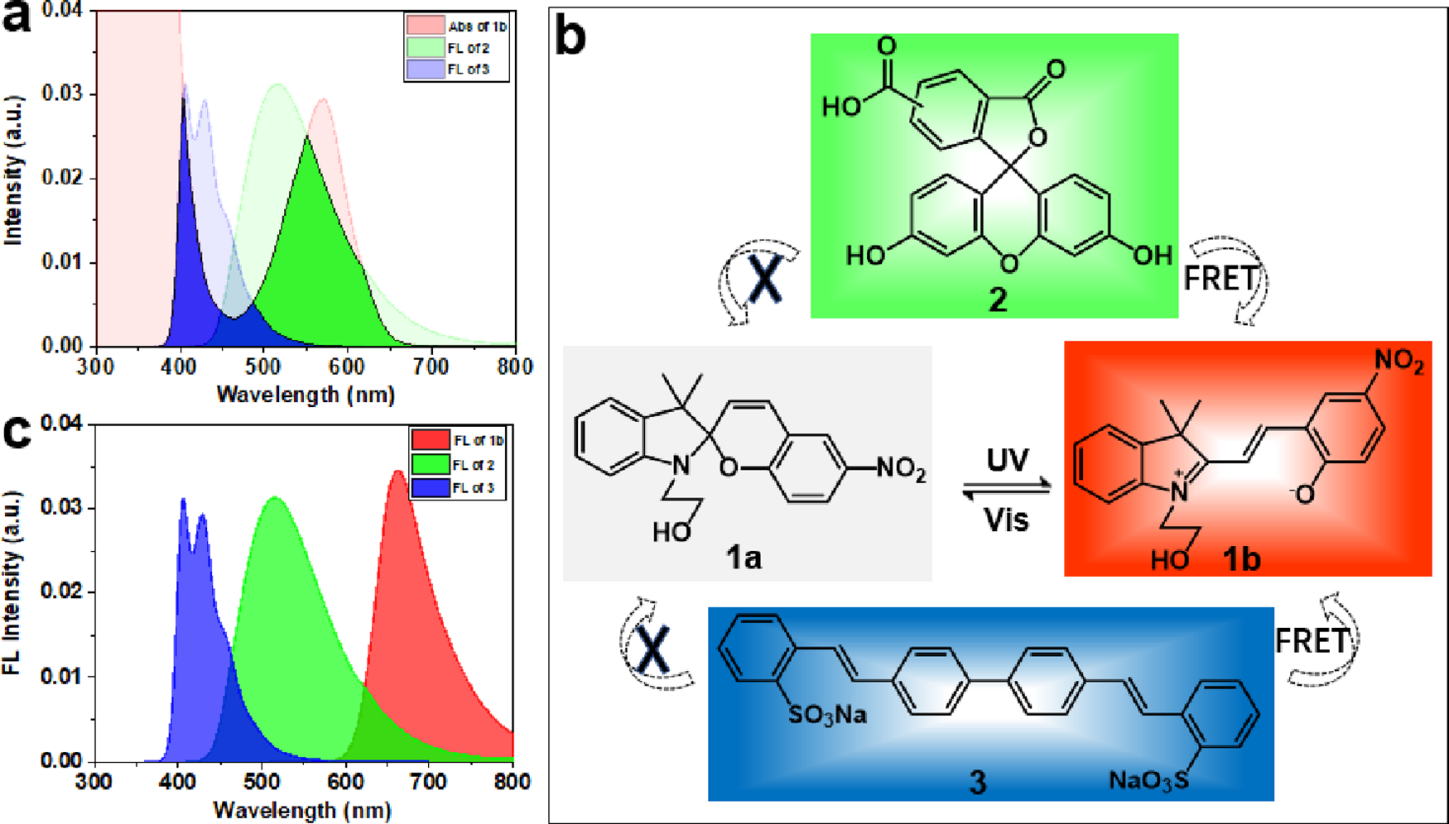
(a) The spectral overlap between the fluorescence spectrum of **2** and **3** and the absorption spectrum of **1b**. (b) Isomerization of **1a** and **1b** under UV (365 nm, 10 mW/cm^2^) and vis (470 nm, 15 mW/cm^2^) irradiation and an illustration of the dynamic FRET process. (c) The fluorescence spectra of **2**, **3**, and **1b**.

The structures and absorption, and fluorescence spectra of the ring-opened form of spiropyran (**1b**), 5(6)-carboxyfluorescein (**2**), and disodium 2,2′-[biphenyl-4,4′-diyldiethene-2,1-diyl]dibenzenesulfonate (**3**) are displayed in Figure S1. When the spiropyran is in the ring-opened isomer **1b** that emits red fluorescence, there are two obvious absorption bands between 500 and 700 nm and below 500 nm, which overlap largely with the emission spectra of fluorophores **2** and **3**, respectively (Figure 1a). Effective FRET channels can be established when the physical distance between the donor and the acceptor is appropriate (in this case, between **1b** and **2**, and **1b** and **3**). When the spiropyran is in the non-fluorescent ring-closed form (**1a**), there is no FRET possible between **2**, **3**, and the spiropyran (Figure 1b). FRET can be generated and dynamically modulated by changing the ratio of **1a** and **1b** through external light stimuli. As shown in Figure S2, with increased time of visible (470 nm) irradiation, the fluorescences of **2** (Figure S2c,d) and **3** (Figure S2a,b) almost remain unchanged. But for **1b**, increasing the time of 470 nm irradiation closes the spiropyran, and the fluorescence intensity decreases due to the loss of fluorescent **1b** (the change in fluorescence intensity with irradiation time fits a first-order process (Figure S2e,f,g)). Thus, in a FRET process using these three materials, with photochromic **1** used as the switching/dynamic component and **2** and **3** used as the static counterparts, tunable photoresponsive fluorochromic materials can be generated.

**Figure 2:**
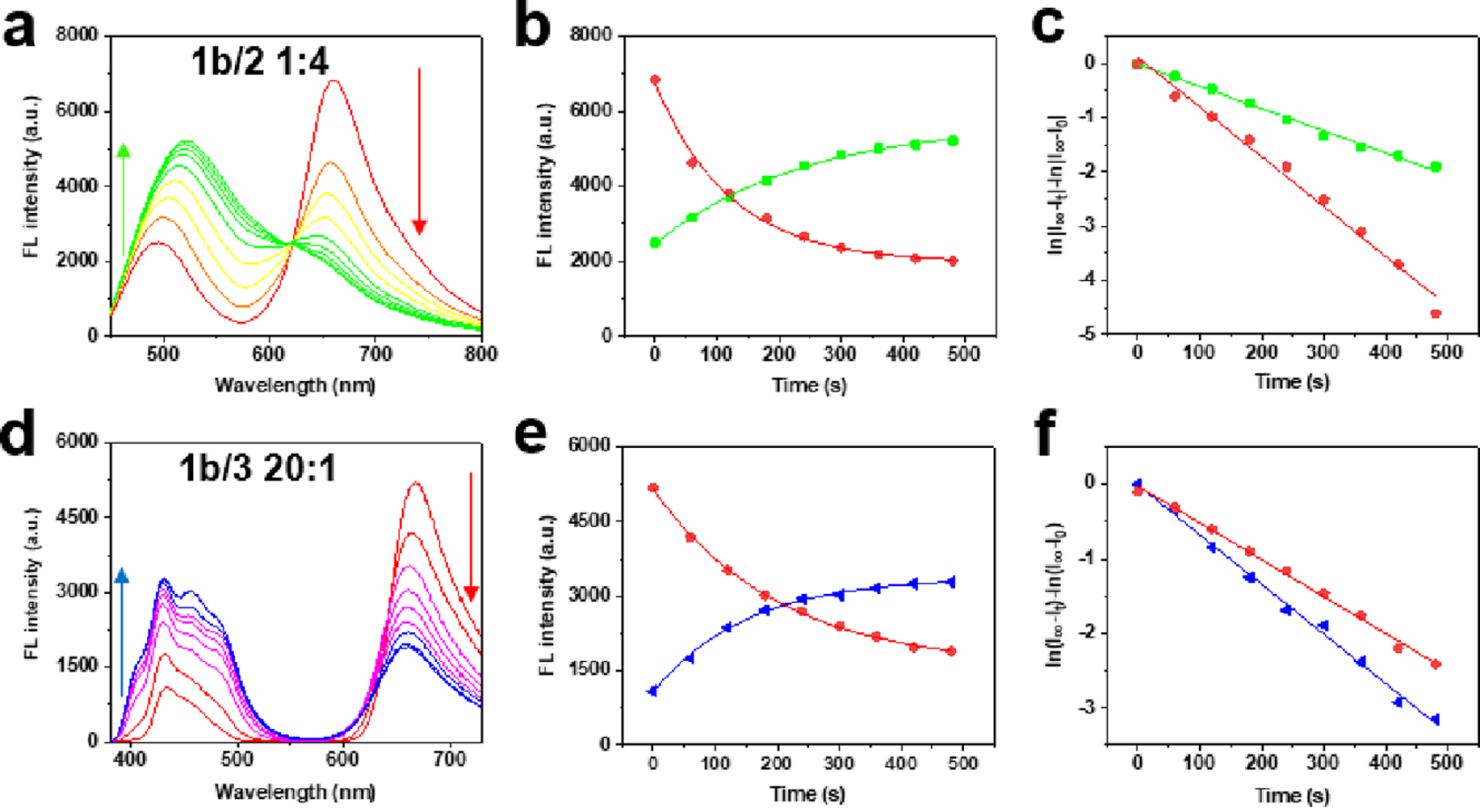
(a) The fluorescence spectra and (b) the fluorescence intensity changes of the red (the maximum peak intensity of each spectrum from panel a in the range of 651–659 nm) and the green (the maximum peak intensity of each spectrum from panel a in the range of 495–521 nm) bands for the mixed solution of **1b** and **2** with a ratio of 1:4 upon different times of 470 nm (15 mW/cm^2^) light irradiation. (c) The first-order kinetic fitting of the fluorescence intensity changes of the red and green bands from panel b. (d) The fluorescence spectra and (e) the fluorescence intensity changes of the red (the maximum peak intensity of each spectrum from panel d in the range of 658–667 nm) and blue (the maximum peak intensity of each spectrum from panel d in the range of 429–432 nm) bands for the mixed solution of **1b** and **3** with a ratio of 20:1 upon different times of 470 nm (15 mW/cm^2^) light irradiation. (f) The first-order kinetic fitting of the fluorescence intensity changes of the red and blue bands from panel e.

Figure 2 shows the typical spectroscopic changes observed during the FRET process. Before 470 nm exposure, the energy transfers from donors (**2** and **3**) to the acceptor (**1b**) and the emissions from the donors are limited. However, upon increasing the 470 nm exposure time (increasing the concentration of the ring-closed form **1a**), the emission peaks in the green and blue bands increase, but the emission peaks in the red bands decrease (due to the decreasing concentration of fluorescent **1b**). The reason is that isomerization from **1b** to **1a** decreases the FRET efficiency that is tightly correlated to the acceptor concentration (**1b**), and accordingly, the donor emissions are slowly switched on and the acceptor emissions are suppressed. Here the optimized FRET efficiency (in the blue or green bands: based on the decreased FL intensity/the initial FL intensity) was calculated.^9^ From Figure 2 and Figures S3 and S4, when the molar ratio of **1b** to **2** was 1:8, 1:4, and 1:2, the FERT efficiency reached 42, 57, and 70%, respectively, and when the molar ratio of **1b** to **3** was 10:1, 20:1, and 40:1, the FERT efficiency can reach 63, 67, and 70%, respectively, so the FRET efficiency can be tuned by changing the spiropyran/fluorophore ratio.

Figure 2c,f presents the kinetic plots for the fluorescence intensity changes (in the blue, green, and red bands) with different times of 470 nm light irradiation according to the calculation method described and the given formula (details are in the Supporting Information).^33−36^ In the system of FRET between the spiropyran and fluorophores with matched emission bands, the kinetics are related to four parameters: the initial concentration of the spiropyran, the ratio between the spiropyran and the fluorophores, the excitation light intensity, and the UV/vis irradiation time. Here the initial concentration of spiropyran and the excitation light intensity when energy transfer occurs are fixed, and the ratio between the spiropyran and the fluorophores and the UV/vis irradiation time are changed. The fluorescence changes of both the energy donors and acceptor are fitted well by exponential equations, and the linear relationships demonstrate that they are first-order reactions as shown in previous works.^18,37,38^ The fluorescence intensity and kinetic analysis of mixtures composed of **1b** and **2**, and **1b** and **3** in various ratios were evaluated with differing 470 nm light irradiation times (Figures S3 and S4). According to the reaction order and kinetic rates of different blending ratios (presented in Table S1), they are comparable to the pure spiropyran, thus the isomerization between **1a** and **1b** is the determining step in the dynamic FRET process. Also we noticed that compared with the kinetic rate constant *k*_1_ (0.00739 s^–1^) for pure spiropyran, *k*_1_’s (0.00926, 0.00919, and 0.00979 s^–1^) for the mixtures of **1b** and **2** with different ratios are slightly higher, while *k*_1_’s (0.00316, 0.00427, and 0.00484 s^–1^) for the mixtures of **1b** and **3** with different ratios are slightly lower (Table S1), which could be rationalized by the change in polarity of the different mixtures. With the isomerization of **1b** to **1a**, the polarity decreases because the ring-opened form **1b** is more polar than the ring-closed form **1a**. The polarity of **2** is lower than **3** (**3** contains ionically charged groups), which presumably facilitates the formation of **1a**, so the *k*_1_ for the mixture of **1b** and **2** increases and the *k*_1_ for the mixture of **1b** and **3** decreases.

Upon exposure to 365 or 470 nm light, the fluorescence peaks in the spiropyran and fluorophore mixtures can be made to fluctuate reversibly, with the fluorescence emissions of the donors and acceptors switched on and off repeatedly by the ″forward and backward″ FRET process. As shown in Figure S5, after 20 cyclic irradiations, there are no apparent changes in the fluorescence intensity of peak and valley values from the fluorescence spectra, indicating the outstanding stability and reversibility of the constructed photoresponsive fluorochromic materials.

### 2.2 Fabrication of Flexible and Fluorochromic Polymer Microarrays

Herein inkjet printing, that can selectively print a solution at desired positions on substrates in accordance with predesigned patterns, was used to fabricate polymer microarrays on flexible PDMS films under ambient conditions. By mixing part A and part B of SYLGARD 184 Silicone Elastomer in a mass ratio of 10:1 and heating, the PDMS film could be obtained after solidification (Figure S6). Photographs show the high transparency of the PDMS film, which is crucial for later optical applications. Also, the flexibility of the PDMS is excellent; whether the film is bent, folded, twisted or otherwise shaped, it can completely return to its original state. This lays a solid foundation for subsequent deformation applications on curved and irregular surfaces (Figure S7).

**Figure 3:**
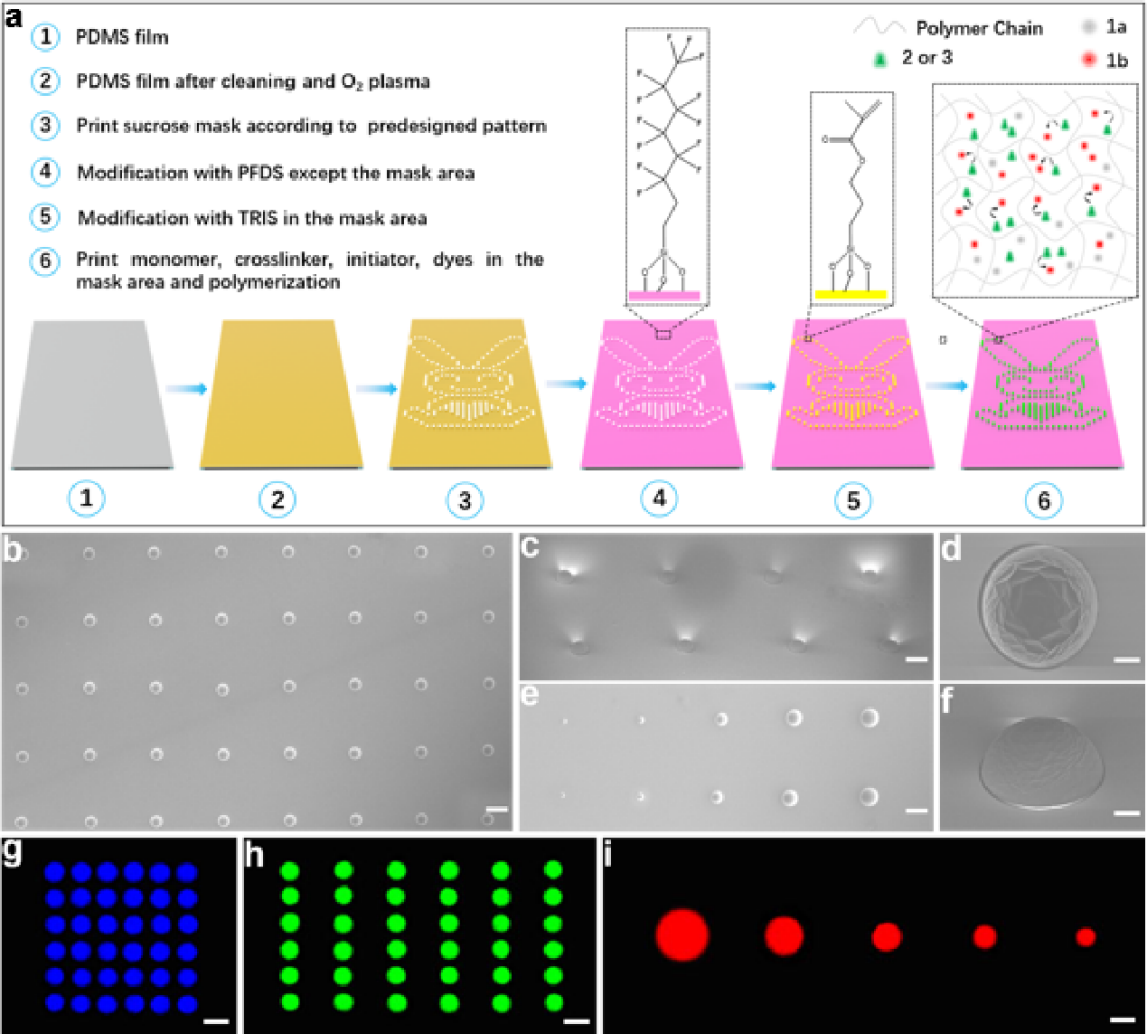
(a) Illustration of the preprocessing of the PDMS substrate and fabrication of the polymer microarray. (b–f) SEM images of the prepared dye-doped polymer microarray: (b) top view (scale bar: 200 μm); (c) side view (scale bar:100 μm); (d) top view of a magnified image of one polymer feature (scale bar:10 μm); (e) top view of features printed with different sizes (scale bar:500 μm); and (f) side view of a magnified image of one polymer feature (scale bar:10 μm). (g–i) Fluorescence microscopy images of the polymer microarrays: (g) doped with **3**, (h) doped with **2**, and (i) doped with **1b**. All scale bars are 100 μm.

To allow good spot morphology and stable polymer features on the substrate, the film surface was preprocessed as shown in Figure 3a. The surface was initially temporally masked with sucrose (according to a predesigned pattern) and then coated with 1*H*,1*H*,2*H*,2*H*-perfluorooctyldimethylchlorosilane (PFDS) to generate a fluorous surface. The sucrose mask and unreacted PFDS were removed by washing, and subsequently, the exposed ″demasked″ surface was functionalized with the acrylsilane. At each designed position, after the monomer, cross-linker, initiator, and dyes/fluorophores are printed and polymerization is initiated, the polymer matrix was cross-linked. Since there will be some penetration into the substrate, a 3D covalent network will be generated with the dyes/fluorophores confined and trapped within (Figure S8).^39^ This will provide a very stable environment for functional molecules to withstand subsequent deformable applications. Here the monomer was acrylamide, and the cross-linker was *N*,*N*′-methylenebis(acrylamide). The functional dyes/fluorophores are entrapped into the polyacrylamide 3D gel networks after polymerization. The polyacrylamide is selected as the polymer matrix for the construction of optical microstructures because of its high compatibility and excellent printability. Polyacrylamide also exhibits high optical transparency in the visible region, which would avoid optical losses caused by energy transfer between doped photochromic materials and the host matrix, permitting us to construct photonic devices with high performance. Benefiting from the high versatility of the inkjet printing, **1**, **2**, and **3** or other dyes and functional materials can be doped in the polymer features together or separately according to different applications. Finally, large-scale pixelated functional microarrays are precisely integrated to form the predesigned pattern.

The SEM images of the features across the microarray showed that they are arranged regularly. Each feature exhibits a hemispherical morphology with a circular boundary, which will minimize undesired optical scattering and effectively confine emission to inside the feature (Figure 3b,c), demonstrating that acrylamide is a good choice for the printing. There are some slight folds on the surface, presumably caused by shrinkage during polymerization (Figure 3d,f). When **2**, **3**, and photoresponsive **1b** were incorporated into the features, clear and bright blue, green, and red emissions can be observed from the fluorescence optical microscope images (Figure 3g,h,i). The emissions are uniform across each feature, indicating that the dyes are well dispersed. Since, in these flexible polymer microarrays, the functional dyes and fluorophores are entrapped in the 3D cross-linked polyacrylamide gel networks, they can migrate into similar microcavities and domains in gels where the free volume is large and maintain their inherent properties.

To increase the feature density and brightness of the dye-doped polymer microarrays, the smaller the feature size and spacing are, the better. The size and the spacing distance can be rationally modulated over a wide range by varying the corresponding parameters in the software (Figures S9 and S10); e.g., by adjusting the number of drops, we can modulate the size, and by adjusting the dot pitch, we can modulate the spacing. Here, the minimal size of one feature was about 80 μm and the minimal spacing between adjacent features was about 30 μm, giving a pattern resolution of 230 pixels per inch (ppi) (Figure 3g) according to the formula ρ = *L*/(*d*1 + *d*2) (ρ: the density, *L*: unit length, *d*1: diameter, and *d*2: distance between adjacent dots), which is comparable to the literature.^40,41^ Thus, it was an ideal building block for the production of desired and ordered pixel microarrays with high packaging density due to the precise positioning of inkjet printing. Also, different sizes of features can be integrated into one substrate (Figure 3i). The printing process is controllable and reproducible, which is conducive for constructing high-quality microstructures on a large scale. What’s more, the microarray can be printed well on different substrates, indicating that the printed microstructure has high compatibility and universality of diverse substrates (Figure S11), so this process could unlock a wide application scope for most dyes and functional material solutions on a variety of substrates.

### 2.3 Tunable Fluorescence from Individual Features

The photoresponsive fluorochromic materials were developed within a polymer microarray format, and the donor and acceptor were entrapped and located within the Förster distance within each feature by virtue of their high concentrations.^42^ Thus, **1**, **2**, and **3** were doped into the features of the polymer microarray, and individual microarray features were analyzed using a microfluorescence system (Figure S12), with each dye-doped feature excited locally with a focused laser beam. The ratio between spiropyran and fluorophores and the UV/vis irradiation time are changed to modulate the emissions of features.

**Figure 4:**
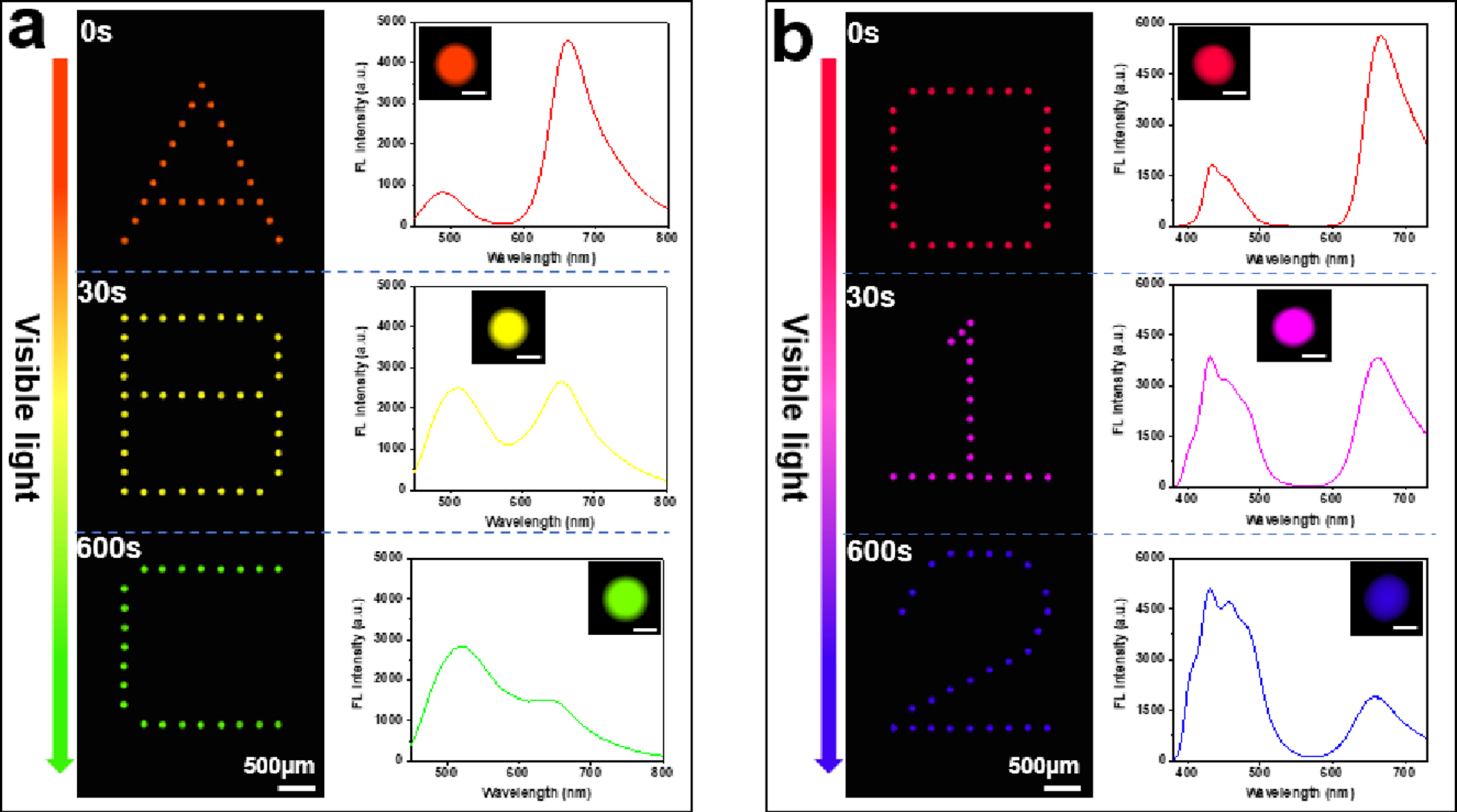
Color generation of the microarrays following different 470 nm (15 mW/cm^2^) light irradiation times and the corresponding fluorescence spectra from an individual feature. (a) Fluorescence images of **2** and **1b** doped microarrays printed as letters ″A″, B″, and ″C″. (b). Fluorescence images of **3** and **1b** doped microarrays printed as numbers ″0″, ″1″, and ″2″. The insets in the spectra are the fluorescence images of the corresponding single feature (scale bar: 50 μm).

For the pattern ″ABC″, the mixture of **1b** and **2** is doped in the microarray (in a ratio of 1:3), and the letters ″A″, ″B″, and ″C″ are irradiated with 470 nm light for 0, 30, and 600 s, respectively. Different fluorescence colors and spectra are obtained as shown in Figure 4a. For the letter ″A″, with no visibe light irradiation, the features emit red fluorescence, attributed to the fluorescent form of the spiropyran (**1b**) giving rise to the peak at 650 nm, because the ring-opened form of spiropyran (**1b**) in the polymer matrix of the letter ″A″ can absorb the green fluorescence of the carboxyfluorescein (**2**) due to the efficient FRET. For the letter ″B″, upon vis irradiation (470 nm, 30 s), the features emit a yellow light with two key peaks centered at 520 nm and 650 nm. This is because both **1a** and **1b** are present, resulting in a partial FRET process and hence dual emissions. For the letter ″C″, after 10 min of 470 nm light exposure, the photochromic reaction is complete to the ring-closed form (**1a**), and hence, the features in ″C″ emit green fluorescence (main peak at 520 nm) originating from the carboxyfluorescein and the suppression of the FRET process (the red fluorescence emission is not possible). With prolonged 470 nm light irradiation from ″A″ to ″C″, the fluorescence intensity from the ring-opened form of spiropyran (**1b**) decreases gradually, while the fluorescence intensity from the donor carboxyfluorescein (**2**) increases, because the FRET efficiency between **1b** and **2** is controlled by the population of **1b** related to the external light exposure time. The optimized FRET efficiency can reach 69% in this microarray.

A microarray doped with the ring-opened form of spiropyran (**1b**) and disodium 2,2′-[biphenyl-4,4′-diyldiethene-2,1-diyl]dibenzenesulfonate (**3**) (in a ratio of 8:1) was similarly fabricated, printing the numbers 0, 1, and 2. Here, the three features (0, 1, and 2) are 470 nm light irradiated for 0, 30, and 600 s, respectively. This gives different emissions (red, magenta, and blue) due to the different FRET efficiencies caused by the different illumination times. For ″0″, the features emit red fluorescence from **1b**, with the fluorescence from **3** well suppressed due to efficient FRET. With 470 nm light exposure for 30 s for ″1″, a stronger emission is obtained from donor **3**, with decreasing emission from the **1b** due to isomerization to **1a**. For ″2″, there is negligible FRET with the fluorescence spectrum dominated by the emission of **3**, leading to distinct color variation across the three features; the optimized FRET efficiency reached 64%.

It is generally agreed that the isomerization of spiropyran can be described by first-order kinetics in solutions^35,36,43^ and in the spiropyran doped gels^44−46^ under UV and vis irradiation except for crystalline polymer matrixes. The isomerization of spiropyran and kinetics in our gel are almost unaffected from our experimental results. The prepared features in the polymer microarrays exhibit switchable and multicolor emissions and similar FRET efficiencies as solutions, indicating that the photoisomerization and FRET process and their kinetics are well-maintained in the polyacrylamide 3D cross-linked gel networks because the free volume is large in the microcavities and domains in the gel and the polymer matrix does not influence the photochromic properties of the doped photoresponsive fluorochromic materials. Thus, the dynamically programmable polymer microarrays could provide an opportunity to construct colorful pixelated panels for information storage as a single feature can function as an individual pixel without the need to combine several dots as subpixels. Due to the optically reconfigurable FERT process, the pixels can emit different colored fluorescence upon defined light exposure.

### 2.4 Flexible Pixelated Panels for Information Storage

The above flexible fluorochromic polymer microarrays can be applied in almost all aspects of information storage, including data (re)writing and erasing, encryption and decryption for anti-counterfeiting, colorful display, and so on. To show the flexibility of the polymer microarrays, photos were taken of the printed patterns on curves with different curvatures (*c* = 1/ρ, ρ is the bending radius of the curve; Figure S13) prepared by 3D printing.

**Figure 5:**
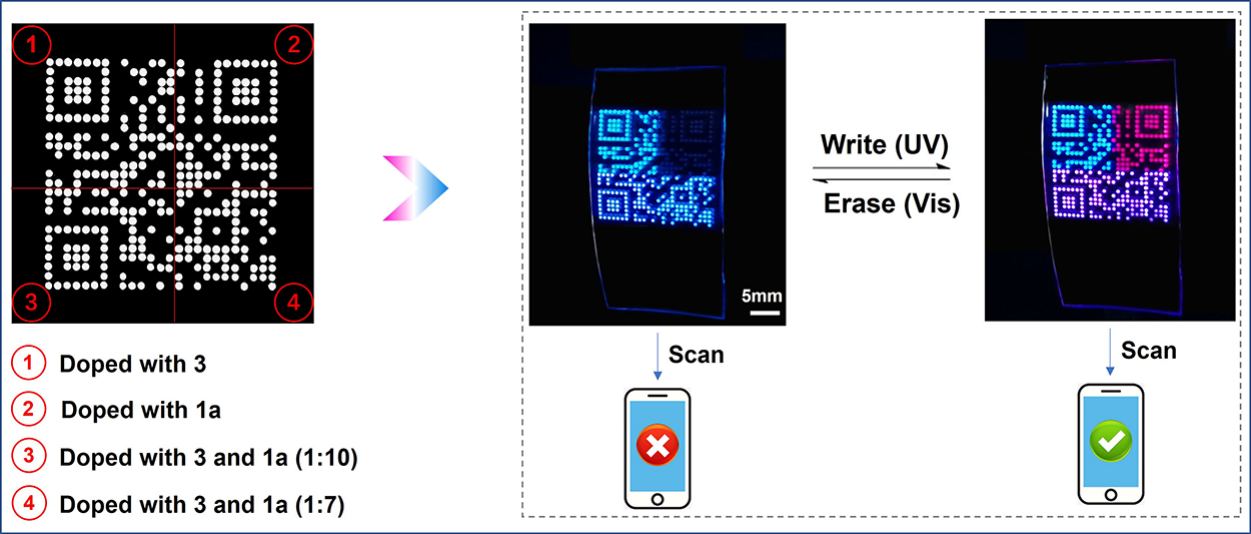
A fluorescent switchable QR code. Left: a black and white fluorescence microscope image of the inkjet-printed QR code (http://www.combichem.co.uk/). Middle and right: images of the printed QR code under different UV (365 nm, 10 mW/cm^2^, 3 min) and visible (470 nm, 15 mW/cm^2^, 8 min) irradiations.

To illustrate the ability of the above polymer microarrays for data storage, a QR code was printed first, with the code divided into four regions, as follows: region ①: only **3** was doped; region ②: only **1b** was doped; and regions ③ and ④: **3** and **1b** are doped together but at different ratios (Figure 5). The whole code will be visible using UV illumination, but the fluorescence of region ② will disappear and the message will be erased by vis illumination due to the isomerization of **1b** to **1a**. Conveniently, the colorful QR code could be scanned on a mobile phone. The relatively slow writing and erasing speed is attributed to the slow isomerization rate of spiropyran (that is not caused by the polymer matrix as even in the solution, the isomerization rate of spiropyran is also slow).^47^ Actually, 60% is completed within 30 s after the UV light is turned on; it takes a long time to reach the equilibrium state later. It was reported that the introduction of electron-donating and electron-withdrawing substituents at the position of 6′ and 8′ into the structure of spiropyran can help to improve its isomerization speed.^48,49^ Exploring the effects of different substituents of spiropyran on its isomerization and energy transfer rate is a follow-up research focus in the polymer matrix gel of this system. It is necessary to find ways to improve the information writing and erasure speed in the QR code. The fatigue resistance of one feature in region ③ of the QR code pattern was examined after 30 consecutive cycles upon UV and vis irradiation (Figure S14). The fluorescence intensity changes in the red and blue bands; both showed good reversibility and negligible decrease, demonstrating that the photoisomerization of spiropyran was robust and the FRET process of the fluorochromic materials proceeded smoothly in the gels. Although the information carried by the colorful code is the same as that carried by the traditional black-and-white code, it also increases interest and makes the visual experience better. With the progress of technology, the information carried by the colorful code will be more abundant and the QR code generated here can be optically controlled with a ″write–read–erase″ operation with a nondestructive readout capability.

**Figure 6:**
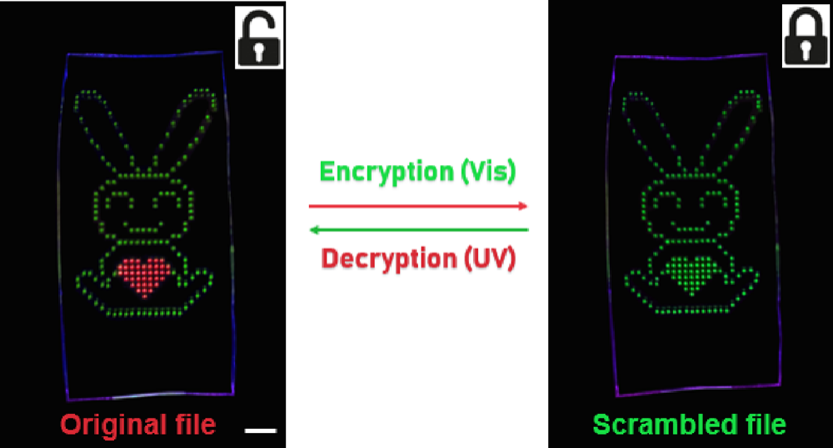
Images of a reversible anti-counterfeiting system using the pattern of a ″rabbit″ with a ″heart″ shape based on the fluorochromic polymer microarrays under UV (365 nm, 10 mW/cm^2^, 2 min) and visible (470 nm, 15 mW/cm^2^, 6 min) exposure. Scale bar: 5 mm.

The demand for high-level information security is growing. Recently, anti-counterfeiting systems based on fluorochromic materials have been developed to increase safety and security.^50,51^ In this study, a reversible anti-counterfeiting platform was constructed based on the programmable fluorochromic polymer microarray. A pattern of a ″rabbit″ with an insert of a heart shape in the middle was printed (Figure 6). The features for the ″rabbit″ were doped with **2** alone, but the ″heart″ was doped with **1b** and **2** together (in the ratio of 1:3). The original and true document is a green fluorescent rabbit with a red fluorescent heart that could be hidden through vis irradiation (470 nm) (i.e., both the rabbit and the heart emitted green fluorescence, and the real information is invisible (encryption)). Only upon UV irradiation is the correct information unlocked and displayed (the ″heart″ showing a notable color and fluorescence change (decryption)). The encryption and decryption processes demonstrated here will also work with a variety of patterns and could be extended to high-security media, such as banknotes and identity documents, with potential in anti-counterfeiting and information security applications.

The uniformity of the features in the microarrays was also evaluated. The fluorescence intensity changes of the green bands for all the 40 features in the heart shape were tested upon UV and visible irradiation to show the uniformity of the printed features. From Figure S15, we can see that the fluorescence change and photoswitching efficiency of 40 features in the heart shape at green bands are similar upon UV and visible irradiation, so the uniformity of the printed features in the microarrays is fine, which will be conducive to the subsequent information storage applications with low signal-to-noise ratios.

**Figure 7:**
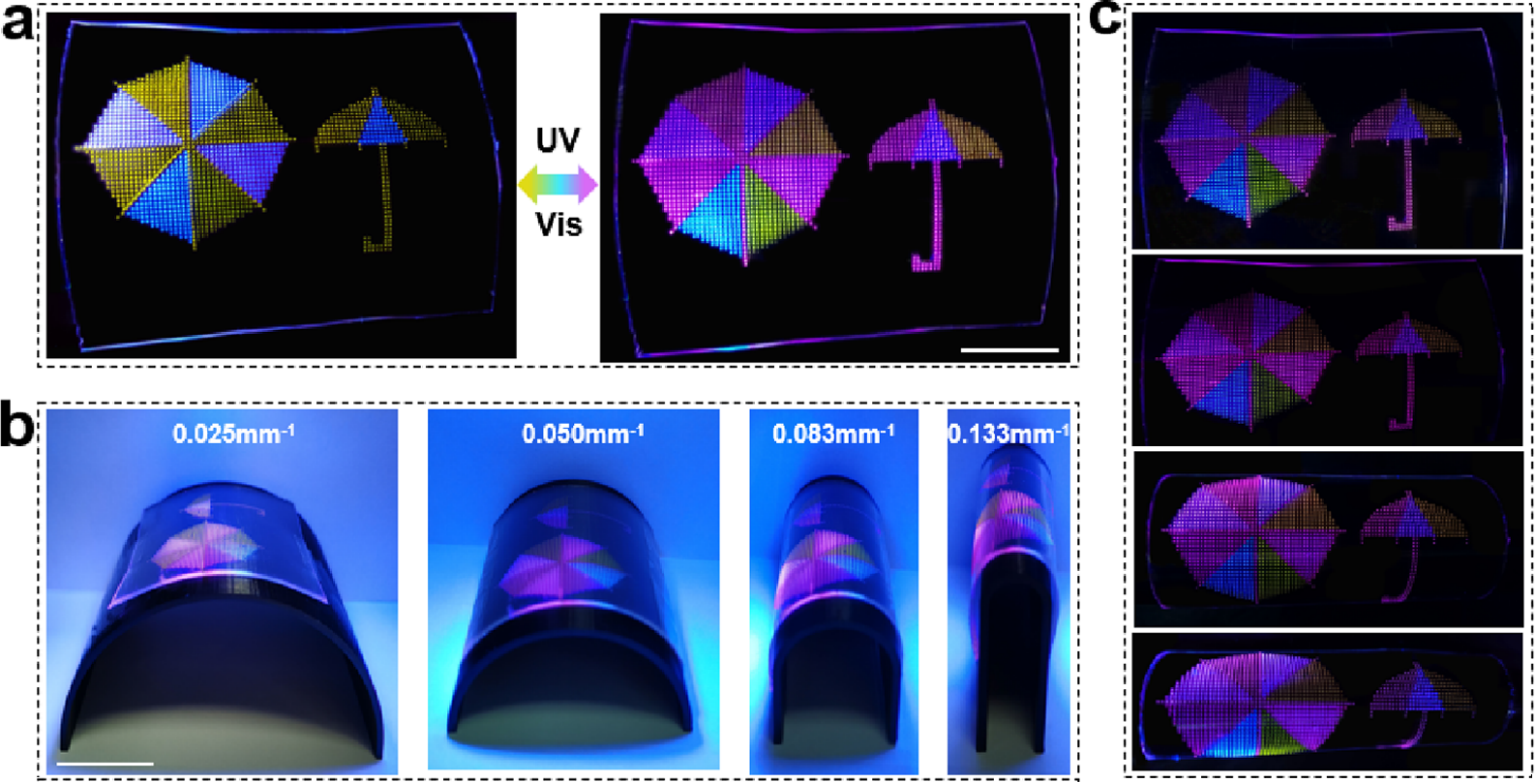
Images of the flexible colorful display of an ″umbrella″ pattern. (a) Under UV (365 nm, 10 mW/cm^2^) and vis (470 nm, 15 mW/cm^2^) irradiation. (b) Side view and (c) corresponding top view of the flexible ″umbrella″ displays under different curvatures. Scale bar: 2 cm.

Another important application of information storage is the display. At present, the lack of flexible pixelated display panels with highly organized emissive geometries impedes the flexible displays in the application of portable and wearable devices.^52^ Here, based on the programmable flexible polymer microarray with dense packing, a vivid, colorful umbrella image is printed. The different regions of the umbrella are printed with different compositions and ratios so that they could change color under UV and vis irradiation (Figure 7 and Figure S16). The display stability under deformations is a crucial issue in the design of flexible display. Thus, the umbrella pattern was analyzed with different curvatures (Figure 7). This shows that there is almost no obvious display difference to the naked eye during cycles of bend and relaxation, demonstrating that the flexible panels formed by pixelated emissive microarrays under deformation maintain a stable imaging performance. Since any image can be considered as a matrix of pixels, different display patterns can be easily generated with each individual feature acting as a pixel. This universal and practical technique allows the fabrication of sophisticated patterns and ″pixelated emissive microarrays″ that will generate colorful panels for outstanding color imaging and flexible displays.

Long-term stability is important for practical applications. Here, using the QR code pattern as an example, the long-term stability of the fluorochromic microarrays was evaluated. After the QR code was stored for 6 months under ambient temperature in the dark, the information can also be written by UV and erased by visible light. Fluorochromic properties were maintained as well as shown in Figure S17. The reason was that the 3D cross-linked polymer matrix was very stable. It can protect the wrapped fluorochromic materials from the bleaching of oxygen, moisture, and oxygen free radicals in the air and increase their stability. Also, the substrate made of PDMS is very stable. The polymer and substrate are polymerized together by cross-linking, which can enhance the stability between the substrate and the microarray features as well. All these confirmed the high robustness and long-term stability of the fluorescent microstructures, which make the application of polymer microarrays in the work as information storage media completely feasible.

## 3 Conclusions

In conclusion, optically programmable polymer microarrays were successfully fabricated on a flexible PDMS substrate through inkjet printing. Photochromic spiropyrans and fluorophores (as energy acceptors and donors for FRET) were incorporated simultaneously into the printed features, endowing the images with intriguing photoresponsive fluorochromic capabilities and sophisticated functions. The photoisomerization process between isomers of spiropyran upon external light stimuli was applied to achieve a tunable acceptor concentration for tailoring the stoichiometric ratio of the acceptor and donor dynamically. As such, every feature can function as a pixel, and the fluorochromic performance can be designed and modulated based on an optically reconfigurable FRET process. The large-scale, flexible, and pixelated microarrays with a resolution of 230 ppi can be used for many aspects of information storage. The marriage of photochromic units with fluorophores into well-assembled microstructures provides a universal strategy to optically manipulate fluorescence behavior and performance at a molecular and microscopic level. The scalable integrated flexible microarrays reported in this work will pave a new avenue for the efficient construction of large-scale colorful information storage devices, with the polymer itself providing a powerful vehicle to protect and enhance the stability of the dyes.

## 4 Experimental Section

### 4.1 Materials

2-(3′,3′-Dimethyl-6-nitrospiro[chromene-2,2′-indolin]-1′-yl)ethanol (spiropyran) (**1**) and tris(trimethylsiloxy)-3-methacryloxypropylsilane were purchased from Fluorochem Ltd. Acrylamide (monomer), *N*,*N*′-methylenebis(acrylamide) (cross-linker), 2,2′-azobis(2-methylpropionitrile) (initiator), and 5(6)-carboxyfluorescein (**2**) were all obtained from Sigma-Aldrich. Disodium 2,2′-[biphenyl-4,4′-diyldiethene-2,1-diyl]dibenzenesulfonate (**3**) was purchased from Generon Ltd. (brand: Neo Biotech). SYLGARD 184 was obtained from Merck Life Sciences. 1*H*,1*H*,2*H*,2*H*-Perfluorooctyldimethylchlorosilane (PFDS) was obtained from Fisher Scientific Ltd. All reagents and solvents were of analytical grade and used as received without any further treatment.

### 4.2 Fabrication of the PDMS Flexible Substrate

The SYLGARD 184 base elastomer and curing agent (mass ratio of 10:1) were mixed together uniformly and gently. To remove air bubbles, the mixture of elastomer and curing agent was placed into a vacuum desiccator for 1 h. The mixture was poured onto a silanized glass surface, evenly spread, and then placed into an oven at 100 °C. After incubating for 3 h, the cross-linked and cured PDMS film was easily peeled off the glass slide and was cut to the desired size as needed for later experiments.

### 4.3 Surface Masking of the PDMS Film

The PDMS film with a moderate size was washed by ultrasonication in water, ethanol, and acetone and dried under a stream of nitrogen. Then the PDMS film was treated with an O_2_ plasma (Diener Plasma system, 50 L/h O_2_, 5 min, 100 W), making the hydrophobic surface hydrophilic. Features (pixels) across the modified PDMS film were masked by inkjet printing five drops of an aqueous sucrose solution (20% w/v; the inkjet ink was the aqueous sucrose solution in this step) to give a predesigned microarray pattern (the equipment is described in the next part). 1*H*,1*H*,2*H*,2*H*-Perfluorooctyldimethylchlorosilane (PFDS, 20 μL) was placed around the periphery of the sucrose printed pattern, and subsequently, the film was sealed in a small box to allow functionalization via the generation of a fluorous surface. After 1 day, the film was washed with water and acetone to remove the sucrose mask and excess PFDS and dried with a stream of nitrogen. Tris(trimethylsiloxy)-3-methacryloxypropylsilane (15 μL) was spread across the top of the dried film and sealed, with the acrylsilane observed to "pooling" into the ″masked″ features. After 24 h, the film was washed with acetone five times, dried under a flow of nitrogen, and kept in a refrigerator until used.

### 4.4 Fabrication of the Emission Polymer Microarrays

The emission polymer microarrays were fabricated through precise spotting on the flexible PDMS substrate using the noncontact sciFLEXARRAYER S5 (Scienion, Germany) inkjet printing system equipped with a PDC 80 Piezo Dispense Capillary (Piezo Systems, Massachusetts, USA; 50 μm nozzle aperture). The printer contained a washing area, a solution absorption area and a printing area, with an XYZ stage, a camera, and a piezoelectric nozzle. It was programmed/run using the software sciFLEXARRAYER (Scienion AG, version 2.19.008.9). The pattern to be printed can be self-designed by highlighting the features in the field setup submenu (one feature can be regarded as one pixel) (Figure S9). In addition, the number of drops printed (the volume of a single drop is 360–440 pL) can be used to modulate the printed feature size.

Here, 50 μL of each reagent solution was transferred into a 384-well microtiter plate and printed (see the Supporting Information). After clicking ″start″ on the software, the whole printing process is automatic and continuous. The solvent will be absorbed automatically to clean the whole machine and nozzle first, and then the nozzle capillary will absorb the dispensing liquid in the microtiter plate and move to the front of camera that is used to visualize the drop volume, shape, stability, and trajectory. These can be adjusted by changing the pulse width (40–60 μs), frequency (450–550 Hz), and piezo voltages (90–120 V) through the software for reliable droplet ejection and good drop morphology. The inkjet ink in this step was the *N*-methylpyrrolidone (NMP) solutions of acrylamide (monomer, 1 M), *N*,*N*′-methylenebisacrylamide (cross-linker, 0.015 M), functional materials (0.001 M, **1b** or **2** or **3** or the mixtures of **1b** and **2** or the mixtures of **1b** and **3** with different molar ratios), and 2,2′-azobis(2-methylpropionitrile) (initiator, 0.005 M). Subsequently, the nozzle moved to the target area and printed the designed pattern driven by piezo. After finishing printing, the nozzle will finally return to the home position. As a part of the automatic printing process, the nozzle was washed with a solvent between each printing cycle. Printing was carried out at 20 °C in an air atmosphere with 50% humidity.

### 4.5 Characterization

UV–vis absorbance spectra were measured using an UV/vis spectrophotometer (Agilent 8453). The fluorescence spectra of the solutions were determined by a spectrofluorophotometer (Shimadzu RF-6000) with a xenon lamp excitation light source with a data interval of 1 nm and a scan speed of 6000 nm/min. All spectral measurements were obtained in a quartz sample cell (with a 1 cm path length) at room temperature. The fluorescence spectra of one feature of the polymer microarray were recorded using an in-house far-field microfluorescence system. The morphology of the polymer microarrays was examined by scanning electron microscopy (SEM, Zeiss Crossbeam 550 FIB-SEM). Fluorescence microscopy images were taken on a Axiovert 200M inverted research microscope (Zeiss) with a 20× objective.

## Supporting Information Available

Molecular structures, and absorption and fluorescence spectra of disodium 2,2′-[biphenyl-4,4′-diyldiethene-2,1-diyl]dibenzenesulfonate (**3**), 5(6)-carboxyfluorescein (**2**), and ring-opened form of spiropyran (**1b**); variation of the fluorescence spectra of solutions of **3**, **2**, and **1b** with different vis irradiation times; the fluorescence spectra and the fluorescence intensity changes for the mixed solutions of **1b** and **2**, and **1b** and **3** with different ratios upon different times of vis irradiation; first-order kinetic parameters for FRET of the **1b**, mixtures of **1b** and **2**, and mixtures of **1b** and **3**; the spectra change of the **1b**, mixtures of **1b** and **2**, and mixtures of **1b** and **3** irradiated repeatedly with UV and vis; the SYLGARD184 Silicone Elastomer Kit used and the molecular structure of PDMS; images of the PDMS film being bent and manipulated and transparency of the PDMS film; schematic illustration of the inkjet printer configuration and printing process; process of designing the pattern to be printed and modulating the parameters using the software sciFLEXARRAYER; bright-field microscopy images of the polymer microarray features on different substrates; the experimental setup for optical characterization; different curvature surfaces fabricated by 3D printing; the changes of fluorescence intensity in red and green band for one feature in region ③ of the QR code pattern upon UV and vis irradiation; the fluorescence intensity changes of the green bands for the 40 features in the heart shape upon vis and UV irradiation; composition and ratio of different regions of the ″umbrella″ pattern; and the long-term stability of the fluorochromic microarrays (PDF)
