# Supplementary material for: Programmable and Flexible Fluorochromic Polymer Microarrays for Information Storage
Source: ACS Appl Mater Interfaces. 2022 May 31;14(23):27107–17. doi: 10.1021/acsami.2c02242 (PMC9204690; doi:10.1021/acsami.2c02242)
Supplement: Supplementary file 1 — am2c02242_si_001.pdf [file am2c02242_si_001.pdf]

Supporting information

Programmable and flexible fluorochromic polymer microarrays for information storage

Hongyan Xia[a,b], Yuguo Ding[b], Jingjing Gong[b], Annamaria Lilienkampf[b], Kang Xie[a], and Mark Bradley[b*]*

[a]Key Laboratory of Precision Electronic Manufacturing Technology and Equipment, School of Electromechanical Engineering, Guangdong University of Technology, Guangzhou, Guangdong 510006, China; EaStCHEM School of Chemistry, University of Edinburgh, Edinburgh EH9 3FJ, United Kingdom.

[b]EaStCHEM School of Chemistry, University of Edinburgh, Edinburgh EH9 3FJ, United Kingdom.

[*]Email: kangxie@gdut.edu.cn; mark.bradley@ed.ac.uk

KEYWORDS

Photo-responsive, fluorochromic, polymer microarray, Förster resonance energy transfer, information storage

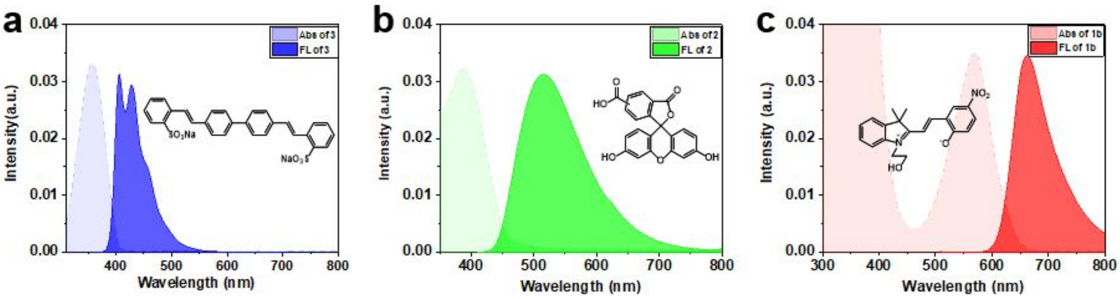

Figure S1. Molecular structures, and absorption and fluorescence spectra of (a). Disodium 2,2'-[biphenyl-4,4'-diyldiethene-2,1-diyl] dibenzenesulphonate (**3**). (b). 5(6)-carboxyfluorescein (**2**) and (c) ring-opened form of spiropyran (**1b**).

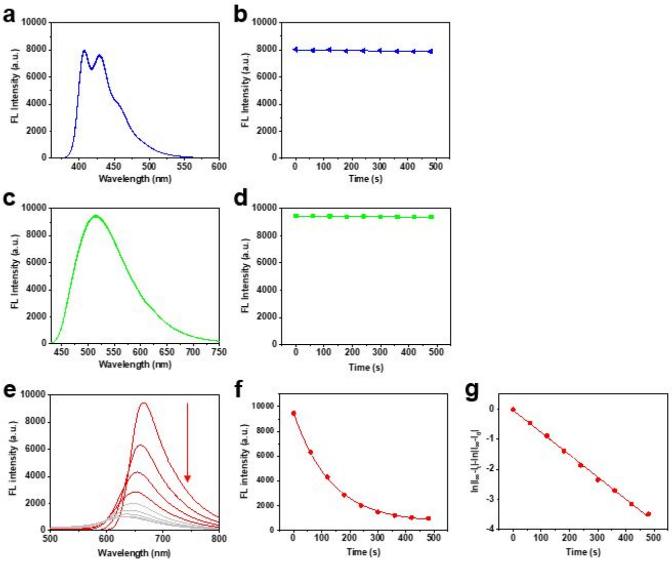

Figure S2. (a), (c) and (e): variation of the fluorescence spectra of solutions of **3**, **2** and **1b** with different 470 nm (15 mW/cm^2) light irradiation times. (b), (d) and (f): variation of the maxima fluorescence peak intensity from (a) (b) and (c) with different 470nm (15 mW/cm^2) light irradiation times. (g). First order kinetic fit of the variation in fluorescence peak intensity with time of 470 nm (15 mW/cm^2) light illumination from (f).

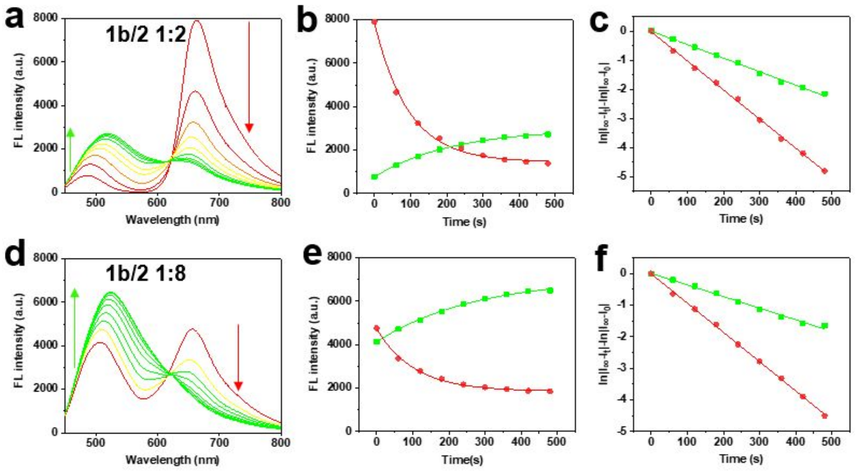

Figure S3. (a) The fluorescence spectra and (b) the fluorescence intensity changes of the red (the maximum peak intensity of each spectrum from (a) in the range of 652-663 nm) and green (the maximum peak intensity of each spectrum from (a) in the range of 487-520 nm) bands for the mixed solution of **1b** and **2** with the ratio of 1:2 upon different time of 470 nm (15 mW/cm^2) light irradiation. (c) The first-order kinetic fitting of the fluorescence intensity changes of the red and green bands from (b). (d) The fluorescence spectra and (e) the fluorescence intensity changes of the red (the maximum peak intensity of each spectrum from (d) in the range of 650-665 nm) and green (the maximum peak intensity of each spectrum from (d) in the range of 507-524 nm) bands for the mixed solution of **1b** and **2** with the ratio of 1:8 upon different time of 470 nm (15 mW/cm^2) light irradiation. (f) The first-order kinetic fitting of the fluorescence intensity changes of the red and blue bands from (e).

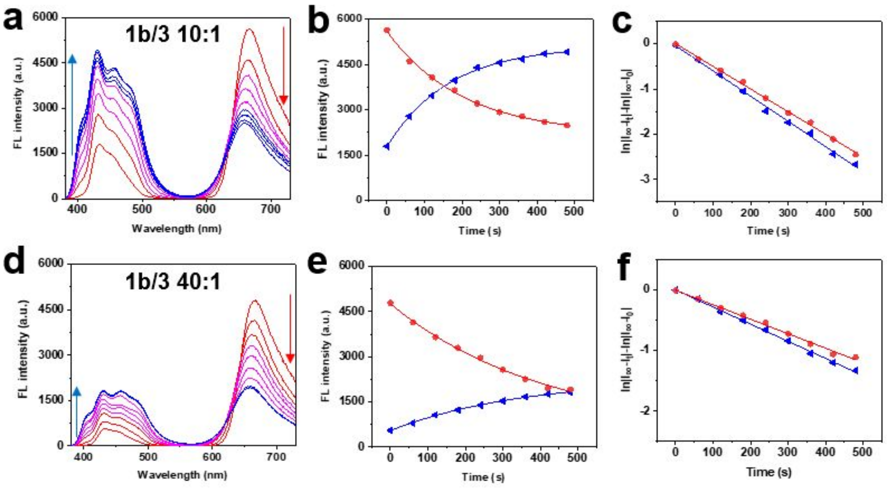

Figure S4. (a) The fluorescence spectra and (b) the fluorescence intensity changes of the red (the maximum peak intensity of each spectrum from (a) in the range of 658-665 nm) and blue (430 nm) bands for the mixed solution of **1b** and **3** with the ratio of 10:1 upon different time of 470 nm (15 mW/cm^2) light irradiation. (c) The first-order kinetic fitting of the fluorescence intensity changes of the red and green bands from (b). (d) The fluorescence spectra and (e) the fluorescence intensity changes of the red (the maximum peak intensity of each spectrum from (d) in the range of 657-664 nm) and blue (430 nm) bands for the mixed solution of **1b** and **3** with the ratio of 40:1 upon different time of 470 nm (15 mW/cm^2) light irradiation. (f) The first-order kinetic fitting of the fluorescence intensity changes of the red and blue bands from (e).

According to the definition of reaction order,

$$-\frac{\mathrm{dc}}{\mathrm{dt}} = kc^n \qquad (1)$$

$$-\frac{dc}{c^n} = kdt \qquad (2)$$

$$-\int_{c_0}^{c_t}\frac{dc}{c^n} = k\int_0^t dt \qquad (3)$$

$$\int_{c_0}^{c_t}\frac{dc}{c^n}dc = -k\int_0^t dt \qquad (4)$$

When n=1,

$$[lnc]_{c_0}^{c_t} = lnc_t - lnc_0 = -kt \qquad (5)$$

When n>0 and n≠1,

$$\frac{1}{n-1}[c^{n-1}]_{c_0}^{c_t} = \frac{1}{n-1}\left[c_t^{1-n} - c_0^{1-n}\right] = kt \qquad (6)$$

According to the formula *$I=2.3\ I_e\,\varepsilon\ \Phi_f\ b\ c$* *(7)*
(Anslyn, E. V.; Dougherty, D. A., Modern physical organic chemistry. University Science Books: 2006.)
$I \propto$ **c** when other parameters kept the same,
If t=0 or t=∞, the initial of MC c_0 and the final concentration of SP (c_∞, the reaction time approaches infinity) are corresponding to the equilibrium emission intensity (I_∞) after taking out the baseline intensity (I_0):

$$c_0 = c_\infty \propto I_\infty - I_0 \qquad (8)$$

At time t,

$$c_0 - c_t \propto I_t - I_0 \qquad (9)$$

$$c_t = c_0 - (c_0 - c_t) \propto I_\infty - I_0 \qquad (10)$$

Substituting c_0 and c_t into the equations *(5)* and *(6)*,

When n=1, for the first order kinetics process,

$$ln\frac{I_\infty - I_t}{I_\infty - I_0} = -kt \qquad (11)$$

Table S1. First order kinetic parameters for FRET of the **1b**, mixtures of **1b** and **2**, and mixtures of **1b** and **3**.

	$\lambda_{max, R}$ (nm)	k_1 (s^{-1})	R-square	$\lambda_{max, G}$ (nm)	k_2 (s^{-1})	R-square	$\lambda_{max, B}$ (nm)	k_3 (s^{-1})	R-square
1b	640-665	0.00739	0.99761	/	/	/	/	/	/
1b/2 1:8	650-655	0.00926	0.99918	507-524	0.00367	0.99139	/	/	/
1b/2 1:4	651-660	0.00919	0.98631	495-521	0.00409	0.99228	/	/	/
1b/2 1:2	652-663	0.00979	0.99864	487-520	0.00464	0.99558	/	/	/
1b/3 40:1	657-664	0.00316	0.99376	/	/	/	430	0.00283	0.99789
1b/3 20:1	658-665	0.00427	0.99501	/	/	/	430	0.00666	0.99475
1b/3 10:1	658-665	0.00484	0.99739	/	/	/	430	0.00558	0.99563

Note: k_1, k_2 and k_3 are the kinetic rate constant of the acceptor (**1b**) and donors (**2** and **3**), respectively.

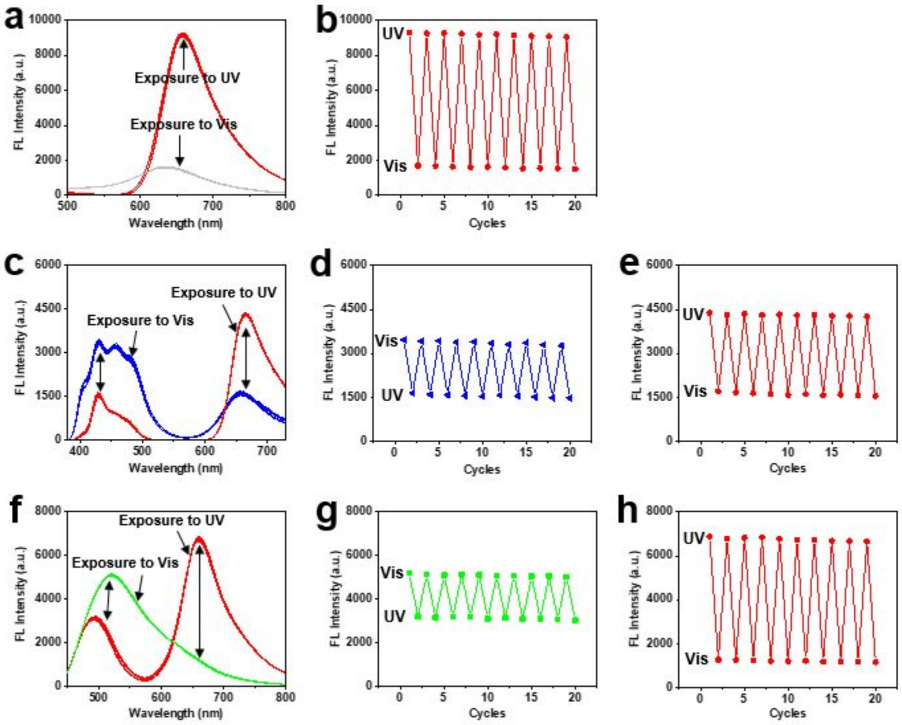

Figure S5. (a) The spectra of the **1b** solution irradiated repeatedly with UV (365 nm, 10 mW/cm^2) for 1 min and Vis (470 nm, 15 mW/cm^2) for 4 min. (b). The rise and fall of the fluorescence intensity at the λ_{max} of the **1b** in the red band from spectra (a). (c) Spectra of the mixed solution with a **1b**/**3** of 15:1 irradiated repeatedly under UV (365 nm, 10 mW/cm^2) for 1 min and Vis (470 nm, 15 mW/cm^2) for 4 min. (d) and (e): The rise and fall of the fluorescence intensity at the λ_{max} of the solution with an **1b**/**3** of 15:1 in the blue and red band. (f) Spectra of mixed solution with an **1b**/**2** of 1:3 and irradiated repeatedly with UV (365nm, 10 mW/cm^2) for 1 min and Vis (470 nm, 15 mW/cm^2) for 4 min. (g) and (h): The rise and fall of the fluorescence intensity at λ_{max} of the solution with an **1b**/**2** of 1:3 in the green and red bands.

Base (part A):

Dimethyl siloxane, dimethylivinyl terminated (CAS No. : 68083-19-2)

Dimethylvinylated and trimethylated silica (CAS No. : 68988-89-6)

Tetra (trimethoxysiloxy) silane (CAS No. : 3555-47-3)

Ethyl benzene (CAS No. : 100-41-4)

Curing Agent (part B)

Dimethyl, methylhydrogen siloxane (CAS No. : 68037-59-2)

Dimethyl siloxane, dimethylvinyl terminated (CAS No. : 68083-19-2)

Tetramethyl tetravinyl cyclotetra siloxane (CAS No. : 2554-06-5)

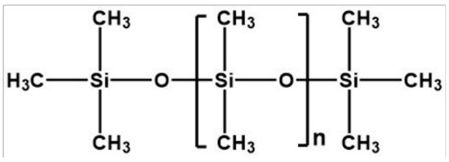

Figure S6. The SYLGARD™ 184 Silicone Elastomer Kit used and the molecular structure of PDMS.

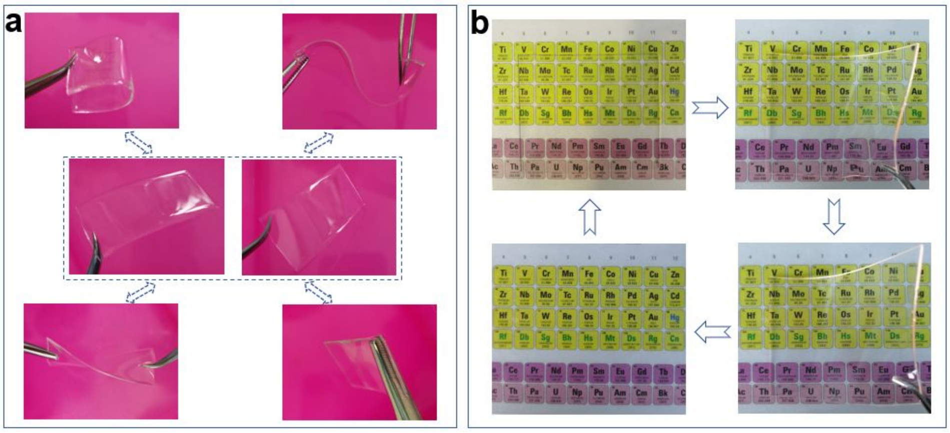

Figure S7. (a) Images of PDMS film being bent and manipulated. (b) Transparency of the PDMS film.

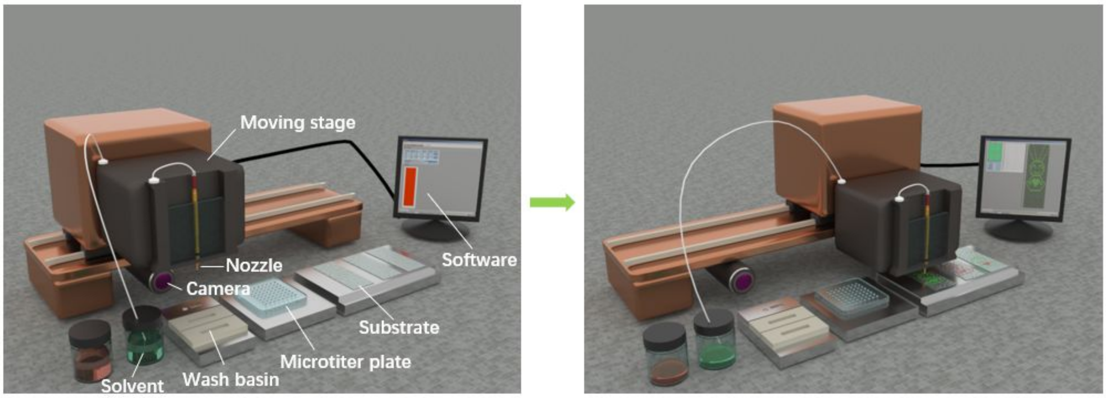

Figure S8. Schematic illustration of inkjet printer configuration and printing process.

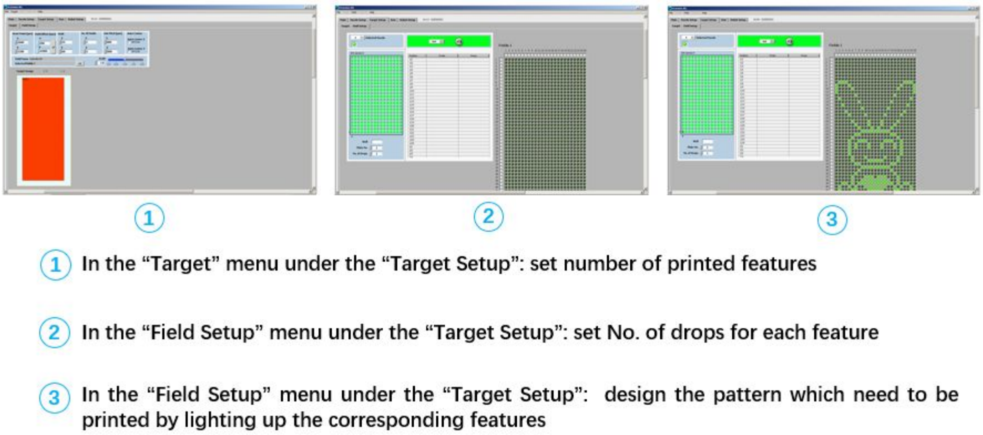

Figure S9. Process of designing the pattern to be printed using the software sciFLEXARRAYER (Scienion AG, version 2.19.008.9).

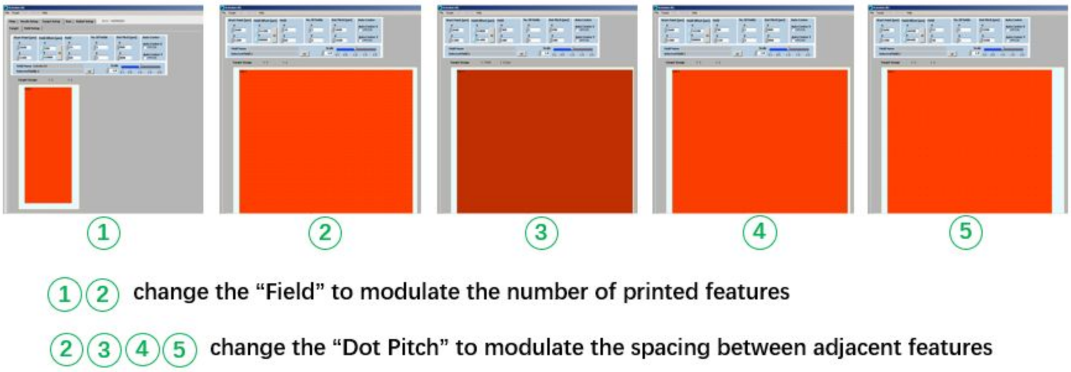

Figure S10. Process of modulating the parameters using the software sciFLEXARRAYER (Scienion AG, version 2.19.008.9).

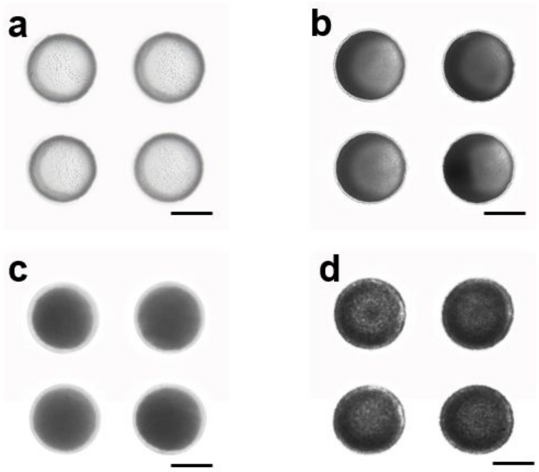

Figure S11. Bright-field microscopy images of the polymer microarray features on different substrates, (a) PDMS, (b) gold, (c) Si, (d) glass. Scale bar: 50 μm.

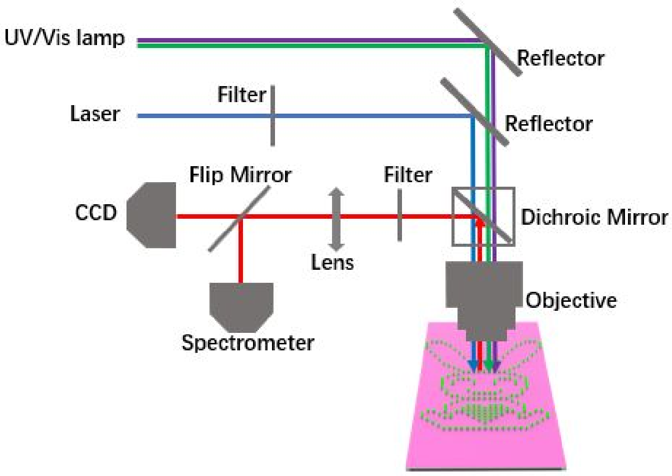

Figure S12. The experimental setup for optical characterization.

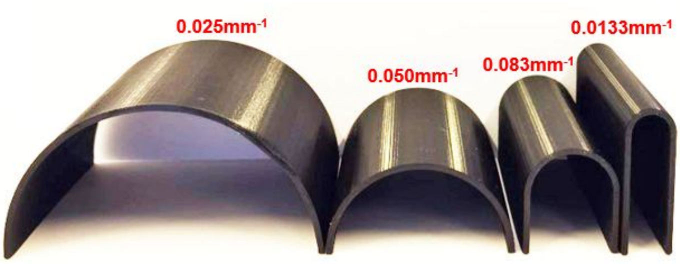

Figure S13. Different curvature surfaces fabricated by 3D printing.

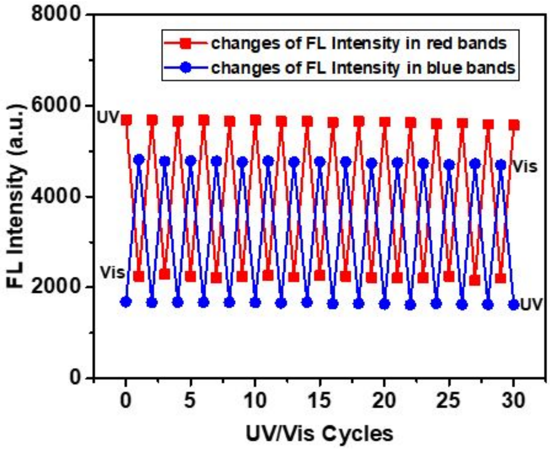

Figure S14. The changes of fluorescence intensity in red and green bands for one feature in the region ③ of QR code pattern upon UV (365 nm, 10 mW/cm^2, 2 min) and Vis (470 nm, 15 mW/cm^2, 6 min) irradiation.

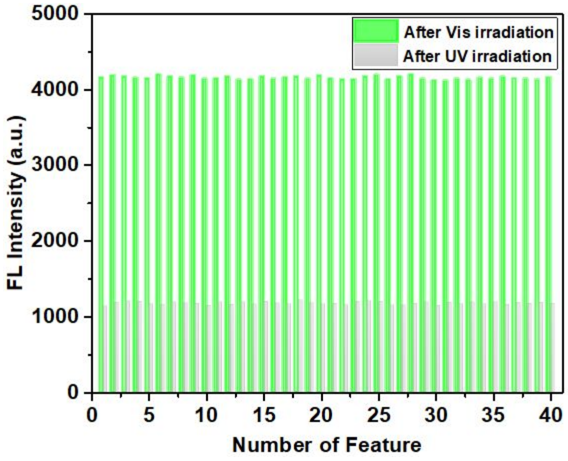

Figure S15. The fluorescence intensity changes of the green bands for the 40 features in the heart shape upon Vis (470 nm, 15 mW/cm^2) and UV (365 nm, 10 mW/cm^2) irradiation.

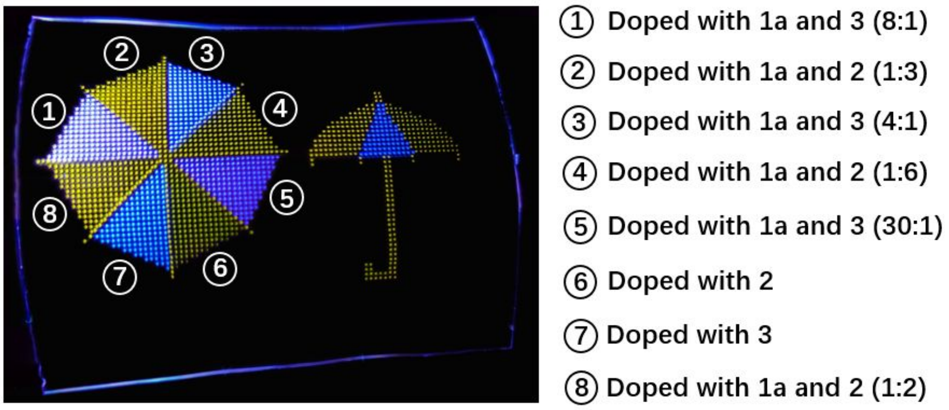

Figure S16. Composition and ratio of different regions of the "umbrella" pattern.

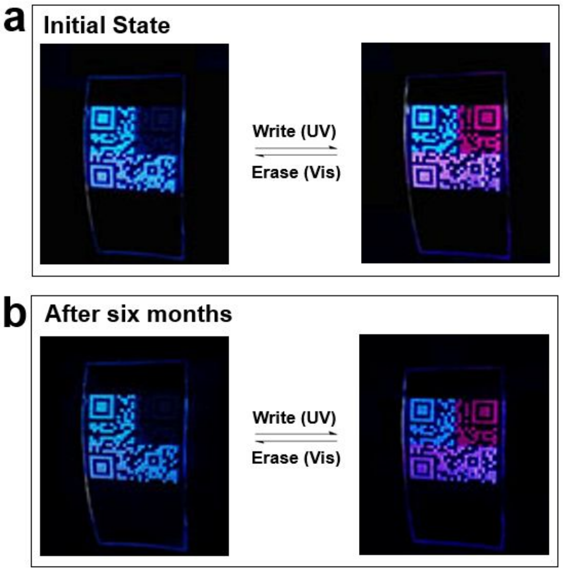

Figure S17. Photographs show the long-term stability of the fluorochromic microarrays: (a) initial state and (b) six months after fabrication.
